# Emergency PCI and TAVR for Acute Myocardial Infarction in Severe Aortic Stenosis Patients

**DOI:** 10.1002/ccr3.72474

**Published:** 2026-04-24

**Authors:** Ruiyuan Lin, Zheng Li, Zhen‐wei Liang, Yuan‐ Yao, Xian‐zi Zeng, Xiao‐mei Gao, Heng‐ Zhang, Zhuo‐Ya Li, Kun Wang, Xiao‐fei Jiang

**Affiliations:** ^1^ The First Clinical Medical College Guangdong Medical University Zhanjiang Guangdong China; ^2^ Faculty of Chinese Medicine Macau University of Science and Technology, Avenida Wai Long Taipa Macau China; ^3^ Zhuhai People's Hospital The Affiliated Hospital of Beijing Institute of Technology, Zhuhai Clinical Medical College of Jinan University Zhuhai Guangdong China; ^4^ The Second Affiliated Hospital of Guizhou Medical University Kaili Guizhou China

**Keywords:** aortic stenosis, bicuspid valve, myocardial infarction, transcatheter aortic valve replacement

## Abstract

Transcatheter aortic valve replacement (TAVR) is a viable alternative for patients with symptomatic severe aortic stenosis. Acute myocardial infarction is a contraindication to TAVR. The efficacy of emergency TAVR combined with percutaneous coronary intervention for patients presenting with acute myocardial infarction, severe aortic stenosis, and cardiogenic shock remains an explorable subject.

**Abbreviations**
AMI: Acute myocardial infarction
AS: Aortic stenosis
CAD: Coronary artery disease
LCA: Left coronary artery
PCI: Percutaneous coronary intervention
SAVR: Surgical aortic valve replacement
TAVR: Transcatheter aortic valve replacement
THV: Transcatheter heart valve

## 1 Introduction

Transcatheter aortic valve replacement (TAVR) has become a pivotal treatment for severe aortic stenosis (AS), with the indications expanding to the low‐risk surgical patients [1, 2]. Meanwhile, the 2025 ESC Valves Conference indicated that for patients aged 70 years or older, the benefits of undergoing TAVR in terms of endpoint events such as mortality, stroke, and rehospitalization are now non‐inferior to those of traditional surgical interventions [3]. However, approximately half of the candidates also have coronary artery disease (CAD) [4], which puts them at a high risk of acute myocardial infarction (AMI) while awaiting the TAVR procedure [5]. The coexistence of severe AS and AMI can lead to poor outcomes, especially during the acute phase. In this case report, we present a successful one‐step emergency treatment involving both percutaneous coronary intervention (PCI) and TAVR for a patient who presented with AMI and severe AS.

## 2 Case History and Examination

**FIGURE 1:**
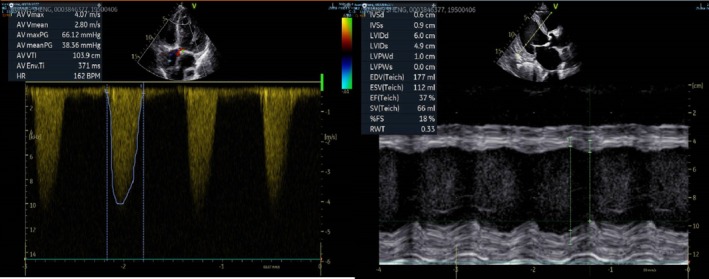
Pre‐procedure transthoracic echocardiography. The mean transaortic gradient of 38 mmHg, the maximum transaortic gradient of 66 mmHg, the outflow tract velocity of 4.1 m/s and the left ventricular ejection fraction was 37%.

A 74‐year‐old male was admitted to our hospital with recurrent dyspnea and hypotension, which is attributed to the severe symptomatic AS. The patient has a history of cerebral infarction and no other underlying conditions. At the time of admission, laboratory tests showed an NT‐proBNP level of 11,500 ng/L and hs‐cTnT level of 0.035 μg/L. Transthoracic echocardiography showed a mean transaortic gradient of 38 mmHg, a maximum transaortic gradient of 66 mmHg, an outflow tract velocity of 4.1 m/s, and the aortic valve area of 0.4 cm^2^. Additionally, the patient's left ventricular ejection fraction (LVEF) was estimated to be 37% (Figure 1), with a New York Heart Association (NYHA) functional classification of Class III.

**FIGURE 2:**
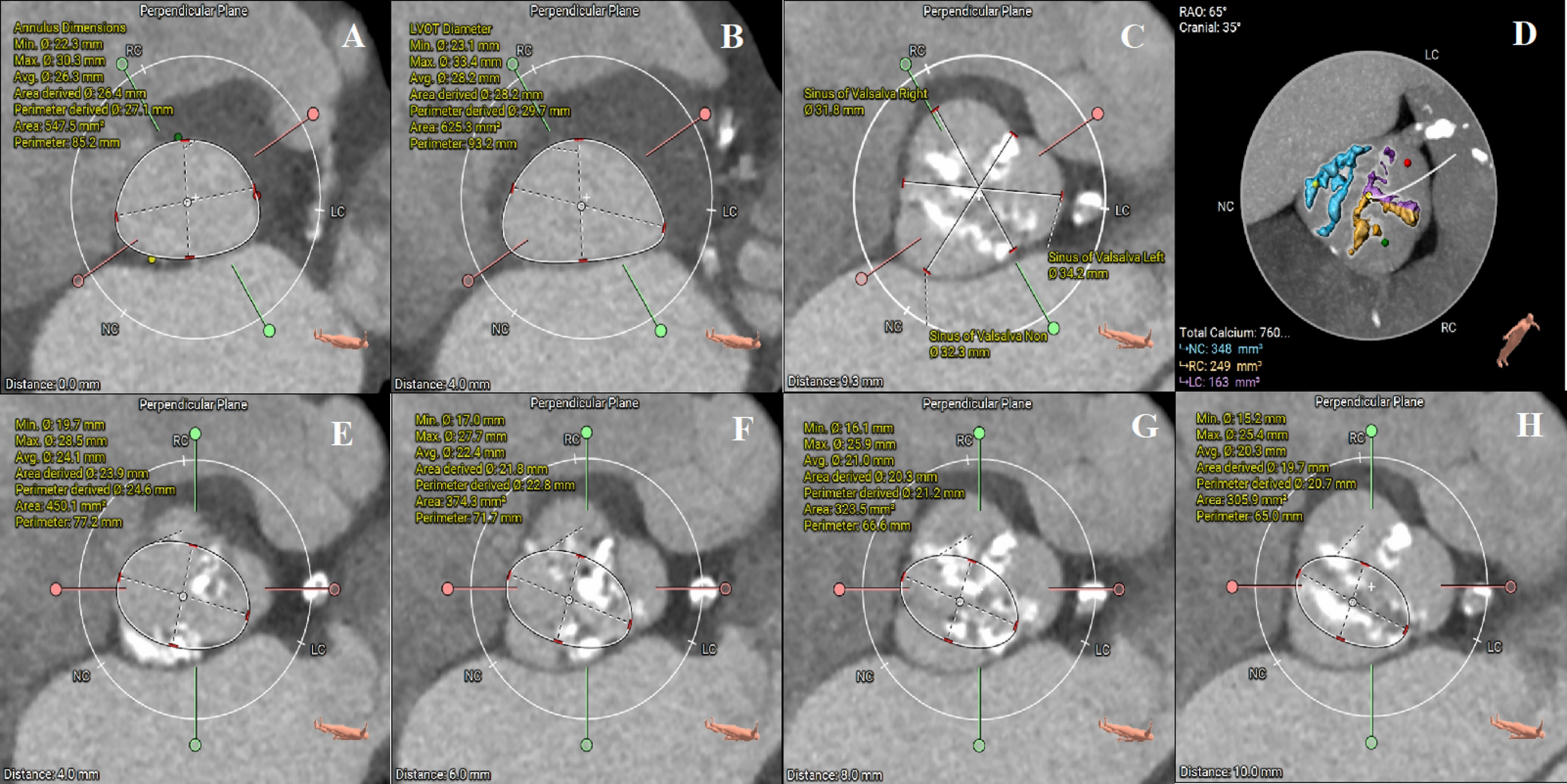
Preoperative computed tomography. (A–D) Tricuspid aortic valve with a 27.1 mm aortic annulus diameter, calcification masses with a total calcium score of 760 mm3. (E–H) Images 4, 6, 8, 10 mm above the annulus, respectively.

Preoperative computed tomography indicated a type‐I bicuspid aortic valve with 27.1 mm aortic annulus diameter, 29.6 mm left ventricular outflow tract and severe calcification between the left sinus and right sinus (Figure 2). The height of the right coronary artery was 19.1 mm and the left coronary artery (LCA) was 14.2 mm. Furthermore, severe stenosis of the LCA has been identified. It was determined to deal with the coronary arteries first. The Society of Thoracic Surgeons score (STS) of this patient was 11.6%, which indicated the high risk in surgical aortic valve replacement (SAVR). Subsequently, a second step for the TAVR procedure was planned.

## 3 Hospitalization Course and Treatment

**FIGURE 3:**
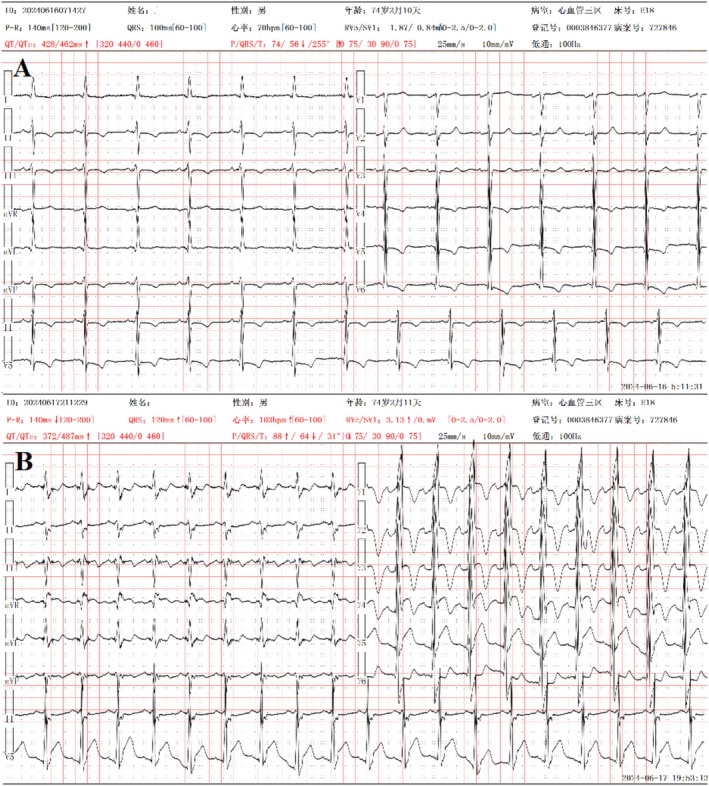
ECG changes: (A) ECG on admission. (B) ECG during an acute episode of chest pain. ECG, electrocardiogram.

During the hospital stay before the coronary procedure, the patient experienced persistent chest pain. An electrocardiogram indicated ST‐segment elevation AMI (Figure 3). Emergency coronary angiography confirmed an occlusion in the proximal anterior descending coronary artery (Video 1), and a drug‐eluting stent was successfully implanted (Video 2). Post‐intervention, the patient's systolic blood pressure remained at 90 mmHg. Additionally, a subsequent transthoracic echocardiogram demonstrated a decline in the LVEF to 20%.

**VIDEO 1:** Emergency coronary angiography. Video content can be viewed at https://onlinelibrary.wiley.com/doi/10.1002/ccr3.72474.

**VIDEO 2:** A drug‐eluting stent was successfully implanted. Video content can be viewed at https://onlinelibrary.wiley.com/doi/10.1002/ccr3.72474.

**FIGURE 4:**
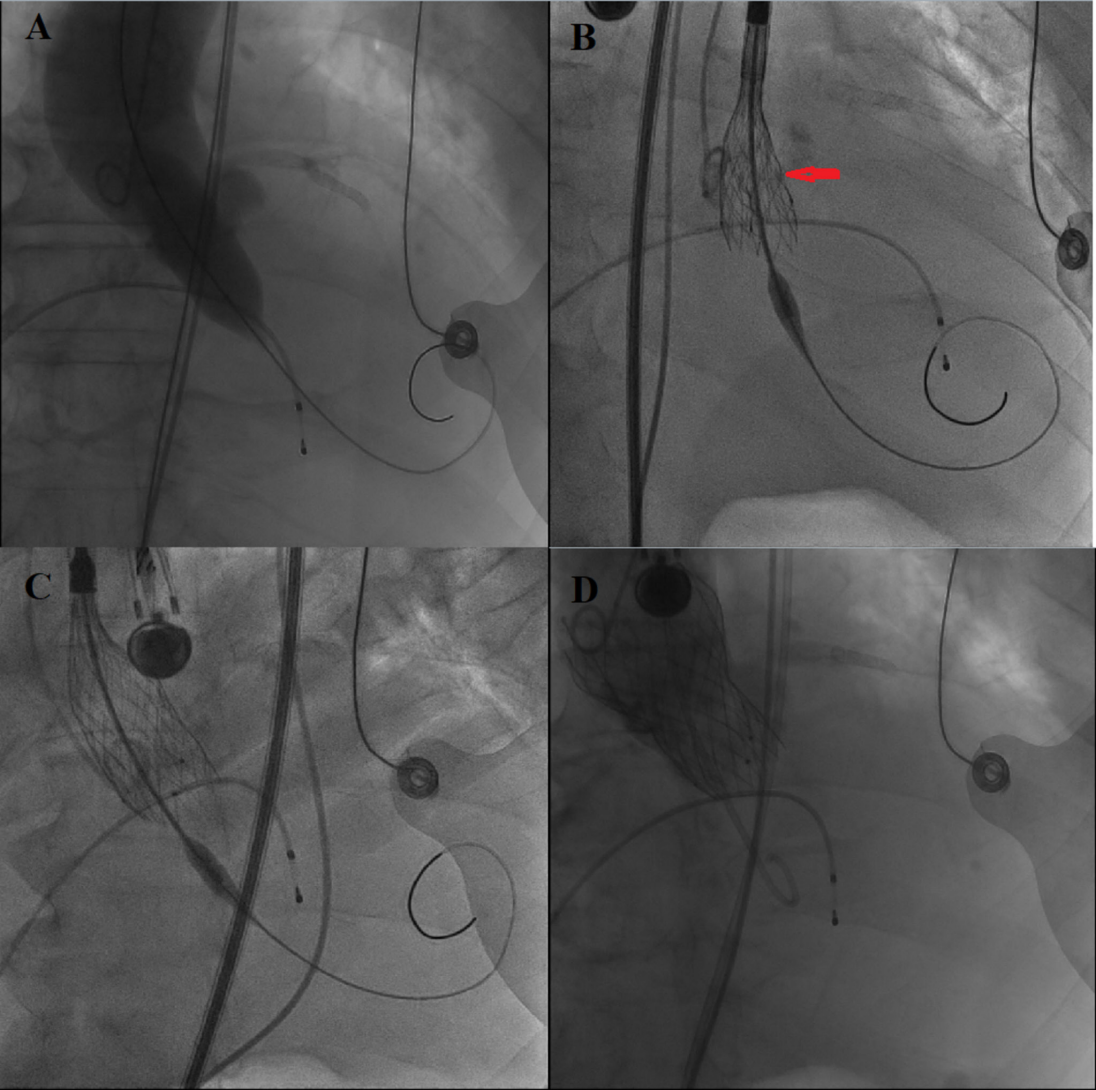
X‐ray images during TAVR. (A) Pre‐dilation with a 20 mm balloon. (B) Under‐expanded valve morphology after the first self‐expandable valve implantation. (C) External chest compression. (D) Post‐dilation with a 25 mm balloon.

**FIGURE 5:**
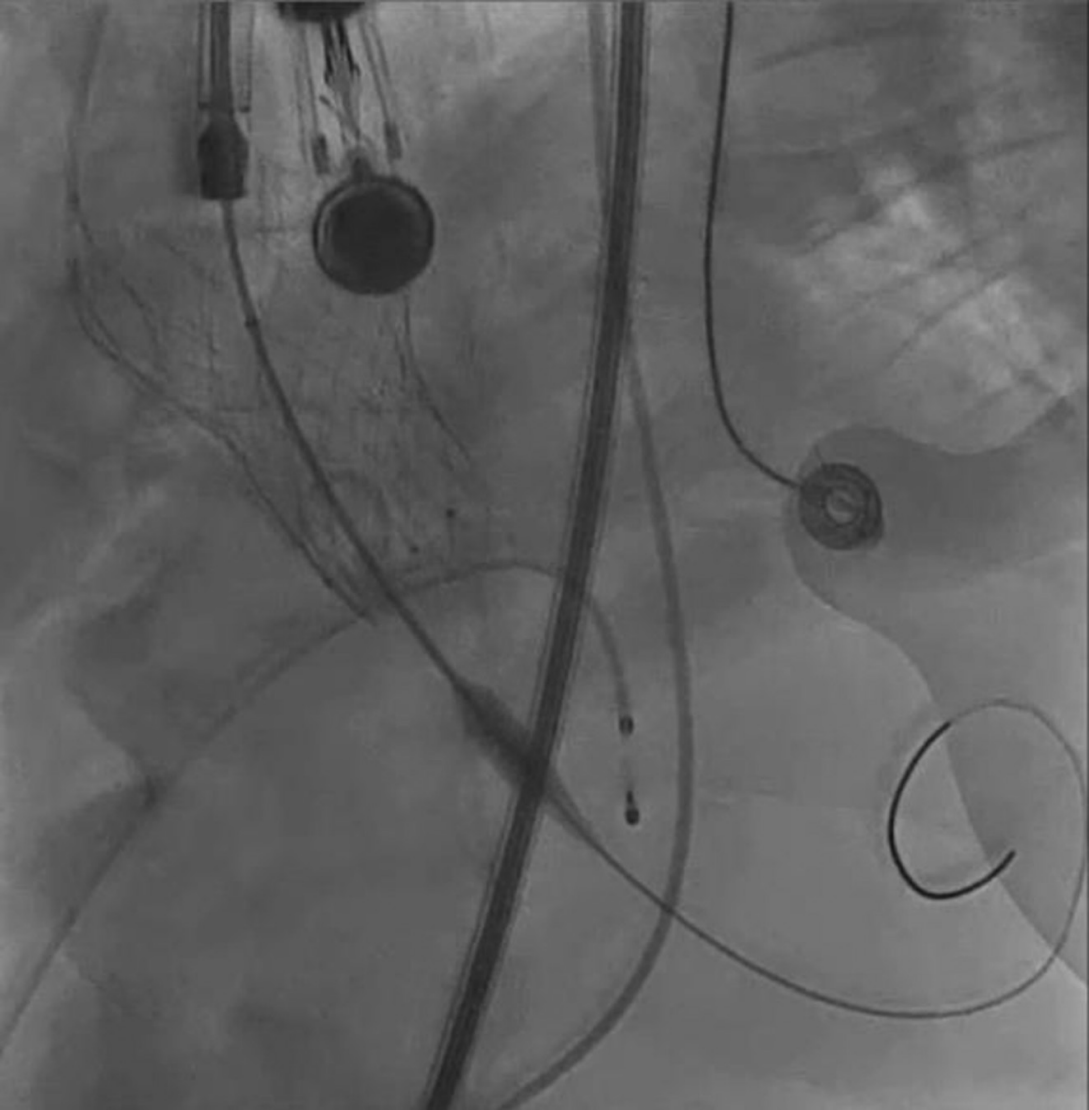
Chest compressions were performed after valve implantation.

After aortography, a 20 mm NuMED balloon (NuMED, Hopkinton, NY, USA) was used to predilate (Figure 4A, Video 3). Subsequently, a 29 mm self‐expandable Venus‐A valve (Venus Medtech Inc., Hangzhou, China) was deployed. However, the configuration of the transcatheter heart valve (THV) was found to be folded (Video 4), and aortography post TAVR revealed moderate paravalvular leakage (Figure 4B). Immediately, the patient experienced cardiac arrest. In response, aside from external chest compression and repeated administration of adrenaline and atropine (Figure 5, Video 5), a 25 mm NuMED balloon was used for repeat post‐dilation (Figure 4C,D). This intervention successfully led to the resumption of normal heartbeat and stabilization of the patient's hemodynamics.

**VIDEO 3:** Predilate with 20 mm balloon. Video content can be viewed at https://onlinelibrary.wiley.com/doi/10.1002/ccr3.72474.

**VIDEO 4:** Transcatheter heart valve was folded during the procedure. Video content can be viewed at https://onlinelibrary.wiley.com/doi/10.1002/ccr3.72474.

**VIDEO 5:** External chest compression. Video content can be viewed at https://onlinelibrary.wiley.com/doi/10.1002/ccr3.72474.

## 4 Conclusion and Results

Following the procedure, the patient was transferred to the cardiovascular intensive care unit and was successfully extubated within 12 h. A follow‐up echocardiogram revealed a well‐seated THV without any signs of paravalvular leak or pericardial effusion. The patient was discharged from the hospital one week after the operation.

## 5 Discussion

SAVR combined with coronary artery bypass grafting (CABG) remains the gold standard for treating patients with aortic valve disease and CAD. An increasing number of studies have confirmed the feasibility and efficacy of TAVR combined with PCI, which is increasingly being recognized as a potential alternative to SAVR+CABG, particularly for high‐risk surgical candidates [6]. The REVASC trial, published in The New England Journal of Medicine in 2024, was a large‐scale, multicenter, randomized controlled trial. It demonstrated that for patients with a small aortic annulus and severe AS, compared to SAVR+CABG, TAVR+PCI yielded comparable results for the primary endpoint—a composite of all‐cause mortality, stroke, myocardial infarction, or rehospitalization. Regarding secondary endpoints, there was no significant difference in all‐cause mortality between the two strategies. The SAVR+CABG group showed advantages in terms of postoperative stroke rates and pacemaker implantation rates. In contrast, the TAVR+PCI group demonstrated benefits in reducing the risks of bleeding and atrial fibrillation, lowering the incidence of postoperative acute kidney injury, and exhibited superior valve hemodynamic performance, quality of life, and recovery [7]. However, it has been observed that such studies exclude patients presenting with acute AMI. In this report, we reported a successful TAVR procedure using a self‐expandable valve in 74‐year‐old male patients who underwent AMI during his hospital stay.

As we know, bicuspid aortic valve poses a challenge for TAVR due to bulky asymmetrical calcification, asymmetrical cups, and commissure fusion [8, 9]. In our case, the patient presented with a severely calcified type 1 bicuspid valve. Consequently, during the deployment of the self‐expandable valves, it folded, obstructing the blood flow to the peripheral circulation. In response to this critical situation, the use of a larger balloon for post‐dilation was crucial to reduce valve folding and expedite the restoration of blood flow. Therefore, this aggressive approach was deemed necessary to mitigate the immediate threat to the patient's life and to ensure a more effective TAVR outcome.

## 6 Conclusion

This article presents a case report of a patient who was admitted with AMI and severe bicuspid AS and successfully underwent a one‐step procedure that included both PCI and TAVR without cardiac assist devices. Despite this combined approach achieving immediate success, further research is necessary to fully comprehend its long‐term outcomes and to inform future clinical practices regarding post‐operative care and monitoring.

## Author Contributions

**Ruiyuan Lin:** conceptualization, data curation, writing – original draft. **Zheng Li:** formal analysis, investigation, visualization. **Zhen‐wei Liang:** data curation, formal analysis, writing – original draft. **Yuan‐ Yao:** data curation, formal analysis, writing – review and editing. **Xian‐zi Zeng:** data curation. **Xiao‐mei Gao:** data curation, formal analysis. **Heng‐ Zhang:** data curation, formal analysis. **Zhuo‐Ya Li:** formal analysis. **Xiao‐fei Jiang:** conceptualization, writing – review and editing. **Kun Wang:** data curation, formal analysis, writing – review and editing.

## Funding

This work was supported by Zhuhai Industry‐University‐Research Collaboration Program (2220004002728) and Guangdong‐Macao Young Talents Two‐Way Exchange Program (207145746053).

## Ethics Statement

The case report and images are reported in accordance with the institutional guidelines in a deidentified manner.

## Consent

Written informed consent was obtained from all relevant patients and their legal guardians for publication of this article, and no ethical conflicts exist.

## Conflicts of Interest

The authors declare no conflicts of interest.

## Data Availability

The data sets supporting the conclusions of this article are included within the article and its additional files.
